# Three-Dimensional-Printed Biomimetic Scaffolds for Investigating Osteoblast-Like Cell Interactions in Simulated Microgravity: An In Vitro Platform for Bone Tissue Engineering Research

**DOI:** 10.3390/jfb16080271

**Published:** 2025-07-24

**Authors:** Eleonora Zenobi, Giulia Gramigna, Elisa Scatena, Luca Panizza, Carlotta Achille, Raffaella Pecci, Annalisa Convertino, Costantino Del Gaudio, Antonella Lisi, Mario Ledda

**Affiliations:** 1 E. Amaldi Foundation, Via del Politecnico snc, 00133 Rome, Italy; eleonora.zenobi@fondazioneamaldi.it (E.Z.); elisa.scatena@fondazioneamaldi.it (E.S.); carlotta.achille@fondazioneamaldi.it (C.A.); 2 Hypatia Research Consortium, Viale I Maggio, 156, Grottaferrata, 00046 Rome, Italy; 3 Institute of Translational Pharmacology, National Research Council, Via Fosso del Cavaliere 100, 00133 Rome, Italy; giulia.gramigna@ift.cnr.it (G.G.); antonella.lisi@ift.cnr.it (A.L.); 4 Violatech srl, Via Kenia 74, 00144 Rome, Italy; luca.panizza@violatech.it; 5 National Centre for Innovative Technologies in Public Health, Istituto Superiore di Sanità, Viale Regina Elena, 00161 Rome, Italy; raffaella.pecci@iss.it; 6 Institute for Microelectronics and Microsystems, National Research Council, Via Fosso del Cavaliere 100, 00133 Rome, Italy; annalisa.convertino@cnr.it; 7 Italian Space Agency, Via del Politecnico snc, 00133 Rome, Italy

**Keywords:** 3D-printed bone-like scaffolds, biomimetics, microgravity conditions

## Abstract

Three-dimensional cell culture systems are relevant in vitro models for studying cellular behavior. In this regard, this present study investigates the interaction between human osteoblast-like cells and 3D-printed scaffolds mimicking physiological and osteoporotic bone structures under simulated microgravity conditions. The objective is to assess the effects of scaffold architecture and dynamic culture conditions on cell adhesion, proliferation, and metabolic activity, with implications for osteoporosis research. Polylactic acid scaffolds with physiological (P) and osteoporotic-like (O) trabecular architectures were 3D-printed by means of fused deposition modeling technology. Morphometric characterization was performed using micro-computed tomography. Human osteoblast-like SAOS-2 and U2OS cells were cultured on the scaffolds under static and dynamic simulated microgravity conditions using a rotary cell culture system (RCCS). Scaffold biocompatibility, cell viability, adhesion, and metabolic activity were evaluated through Bromodeoxyuridine incorporation assays, a water-soluble tetrazolium salt assay, and an enzyme-linked immunosorbent assay of tumor necrosis factor-α secretion. Both scaffold models supported osteoblast-like cell adhesion and growth, with an approximately threefold increase in colonization observed on the high-porosity O scaffolds under dynamic conditions. The dynamic environment facilitated increased surface interaction, amplifying the effects of scaffold architecture on cell behavior. Overall, sustained cell growth and metabolic activity, together with the absence of detectable inflammatory responses, confirmed the biocompatibility of the system. Scaffold microstructure and dynamic culture conditions significantly influence osteoblast-like cell behavior. The combination of 3D-printed scaffolds and a RCCS bioreactor provides a promising platform for studying bone remodeling in osteoporosis and microgravity-induced bone loss. These findings may contribute to the development of advanced in vitro models for biomedical research and potential countermeasures for bone degeneration.

## 1. Introduction

Three-dimensional cell culture systems have transformed biological research, particularly in the field of human health, by offering a more realistic representation of the native tissue microenvironment compared to traditional two-dimensional in vitro cultures [1]. These systems enhance cell–cell and cell–matrix interactions thanks to their spatial organization and biomimetic scaffold surfaces, making them invaluable tools for applications in tissue engineering and regenerative medicine.

In the context of bone tissue engineering, the development of advanced devices is essential for overcoming the limitations of conventional medical prostheses, which can significantly impact the recipient’s quality of life. The possibility to deal with ad hoc structures that are capable of replicating the natural extracellular matrix, a highly complex and functional structure, can effectively support cellular growth, differentiation, and integration with surrounding tissues [2]. Recent advancements in 3D-printing technologies, such as fused deposition modeling (FDM), have enabled the fabrication of scaffolds that mimic the intricate microarchitecture of native bone, including healthy and pathological conditions like osteoporosis [3].

A comprehensive research strategy can then be planned by integrating biomimetic, biocompatible, and bioresorbable scaffolds with dynamic bioreactors, such as rotary cell culture systems (RCCSs), which represent a cutting-edge 3D in vitro set-up to closely reproduce the native bone tissue microenvironment [4]. This experimental approach can be regarded as a promising platform for advanced bone tissue engineering investigations, also supporting the development of innovative therapies for improved treatment strategies and pre-clinical research models.

Bone is a dynamic and hierarchically structured tissue that provides structural support, facilitates movement, and protects internal organs [5,6]. Two different types can be mainly observed: trabecular and cortical bone. Trabecular bone, which accounts for approximately 20% of the skeleton, is characterized by a sponge-like structure with interconnected trabeculae. The porosity ranges from 50% to 90%, significantly influencing mechanical properties, such as modulus and compressive strength [7]. Cortical bone, in contrast, is denser, with a porosity of about 10%, forming the outer layer of bones and providing resistance to bending and torsion. This structure has evolved to respond to a number of different conditions, including mechanical loading, which plays a pivotal role to maintain bone strength through a dynamic remodeling process involving osteocytes, osteoblasts, and osteoclasts.

A substantial modification of external inputs may induce osteoporotic bone condition, a progressive skeletal disorder characterized by bone mass and density loss, compromised bone strength, and microarchitectural deterioration, which significantly increases fracture risk. The disease primarily affects trabecular bone due to its high surface area and metabolic activity. Osteoporotic trabecular bone exhibits notable structural changes, including increased porosity, reduced trabecular thickness, and a loss of connectivity density. These alterations lead to a more fragile, impaired network, reducing its ability to withstand mechanical loads and resist deformation [8]. Replicating such structural features in vitro is essential for creating realistic models to study osteoporotic bone-like tissue behavior and assess scaffold performance under pathological-like conditions.

To better simulate the in vivo bone microenvironment and overcome the limitations of static 2D cultures, dynamic bioreactor systems have been developed. Among them, RCCSs are particularly advantageous due to their ability to generate a low-shear, three-dimensional continuous suspension of bioengineered constructs, enhance nutrient and oxygen diffusion, and mimic in vivo conditions, fostering cell growth, differentiation, and metabolic activity within 3D scaffolds [9]. In a RCCS, scaffolds and cells are maintained in a state of constant free-fall, effectively reducing sedimentation and mimicking some aspects of microgravity. This unique environment supports enhanced cell–scaffold interactions, improved mass transport, and mechanical stimulation, all of which are crucial factors in tissue development, especially in mechanically responsive tissues like bone [10].

Dynamic culture also contributes to mechanotransduction, a process in which cells sense and respond to mechanical cues in their environment, influencing cell proliferation, morphology, gene expression, and matrix production. In bone tissue engineering, such mechanical inputs are especially important, as osteoblast-lineage cells are naturally responsive to mechanical stress. Moreover, when applied to scaffolds with tailored porosity and architecture, such as those designed to mimic osteoporotic traits, dynamic systems allow for a more refined evaluation of how architecture and mechanical cues interact to guide cellular responses.

By integrating these principles, our study aims to validate a platform, combining biomimetic scaffold design and rotary dynamic culture as a realistic and tunable environment for investigating cellular behavior under conditions relevant to osteoporotic bone tissue. Starting from this assumption, we designed and fabricated 3D-printed polylactic acid (PLA) scaffolds using FDM to replicate both physiological and osteoporotic bone architectures. The selected polymer is a common material for bone scaffold investigations, being a biocompatible and biodegradable aliphatic polyester approved by the Food and Drug Administration [11,12]. In addition, PLA exhibits several advantages including the possibility of being obtained from renewable sources and slight piezoelectric properties, making it suitable to design scaffolds for bone tissue applications. The fabricated bone-like scaffolds were thus located within a RCCS bioreactor to be seeded and cultured with osteoblast-like cells (i.e., SAOS-2 and U2OS) in dynamic conditions to be compared with static cultures.

This study’s primary objectives were (i) to evaluate the biocompatibility and functionality of the scaffolds using assays to measure cell proliferation, adhesion, and inflammatory response; (ii) assess how scaffold internal microarchitecture, porosity, and fluid dynamics in the RCCS influence cellular behavior, particularly growth and metabolic activity; and (iii) optimize scaffold design for simulated microgravity conditions, hypothesizing that higher porosity enhances cell growth and metabolic activity by increasing surface area and improving fluid dynamics. The collected findings are expected to highlight the transformative potential of combining advanced 3D cell culture technologies with dynamic bioreactor systems, bridging the gap between in vitro models and in vivo applications. This investigation provides valuable insights into bone remodeling induced by dynamic conditions, paving the way for innovative therapeutic strategies that may address a range of challenges, laying the groundwork for more advanced studies targeting long-term bone cells and scaffolds to treat conditions like osteoporosis more effectively. This integrated platform may serve as a step forward in creating next-generation tissue engineering solutions for both research and clinical applications.

## 2. Materials and Methods

### 2.1. Biomimetic Scaffold Design

The design and preliminary analysis of the proposed scaffolds were performed using Meshmixer software (Meshmixer version 2018, Autodesk, San Rafael, CA, USA). Starting from a previous approach [13], an updated model was prepared to simulate osteoporotic-like trabecular bone tissue. Briefly, biomimetic bone scaffolds were realized to deal with a physiological (P) and osteoporotic (O) model by first subtracting a three-dimensional random cluster of spheres from a box-shaped solid to obtain a porous pattern. Subsequently, specific parameters were set for both the proposed models in terms of pore dimension, pore spacing, trabecular thickness, and trabecular spacing, as summarized in Table 1. The trabecular network was generated using an algorithm based on Delaunay triangulation.

### 2.2. Three-Dimensional Printing Set-Up and Scaffold Evaluation

**Figure 1:**
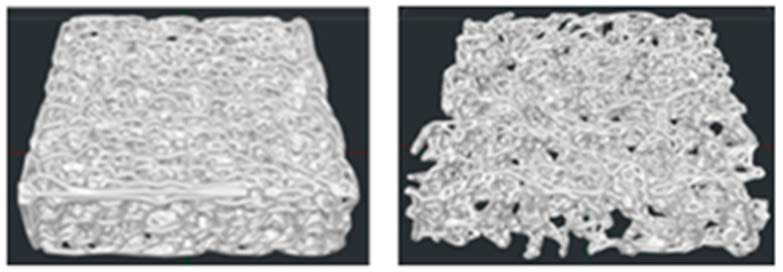
Preview printing of the P scaffold model (**left** panel) and the O scaffold model (**right** panel).

Each scaffold file (Figure 1) was imported in IdeaMaker software (IdeaMaker version 3.6.1, Raise 3D Inc., Irvine, CA, USA) to slice the model and drive the Raise 3D N2 printer (Raise 3D Inc., Irvine, CA, USA). A commercial PLA filament (FILOALFA, Turin, Italy) was extruded at 205 °C through a 0.4 mm-diameter nozzle, the build platform temperature was set at 60 °C, and the positioning accuracy of the 3D printer was 0.0125 mm (XY axis) and 0.00125 mm (Z axis).

Microtomographic analysis (µ-CT) is a non-destructive technique, which allows for obtaining qualitative and quantitative information on the internal structure of the investigated samples. Each scaffold model was therefore analyzed to evaluate the printed microstructure and verify the conformity to the design criteria. For this aim, the microtomograph Skyscan 1072 (Bruker microCT, Kontich, Belgium) was used, powering the X-ray tube at 40 kV and 248 μA, and setting pixel dimensions at 14.65 × 14.65 μm (corresponding to 20× magnification and 180° rotation with a step of 0.45°). The 3D reconstruction of the sample was performed using the NRecon software (Version 1.7.0; Bruker microCT, Kontich, Belgium). The CT Analyzer software (Version 1.16.9.0; Bruker microCT, Kontich, Belgium) was employed to elaborate the collected data and calculate the histomorphometric parameters.

### 2.3. Cell Culture

The human osteosarcoma cell lineages SAOS-2 and U2OS (purchased from the American Type Culture Collection, ATCC, HTB-85 and HTB-96, Rockville, MD, USA) were selected to assess in vitro scaffold biocompatibility, cell growth, and adhesion. Before starting the experiments, cells were seeded on a plastic Petri dish and placed in a humidified incubator at 37 °C and 5% CO_2_. Cells were cultured in high-glucose Dulbecco’s modified Eagle’s Medium (DMEM; Euroclone, Milan, Italy), completed with 10% heat-inactivated fetal bovine serum (FBS; Euroclone, Milan, Italy), 2 mM L-glutamine (Sigma, Darmstadt, Germany), 1.0 unit mL^−1^ penicillin (Sigma, Darmstadt, Germany), and 1.0 mg mL^−1^ streptomycin (Sigma, Darmstadt, Germany). Scaffolds were sterilized with 70% ethanol solution and accurately washed before cell seeding.

### 2.4. RCCS Bioreactor

**Figure 2:**
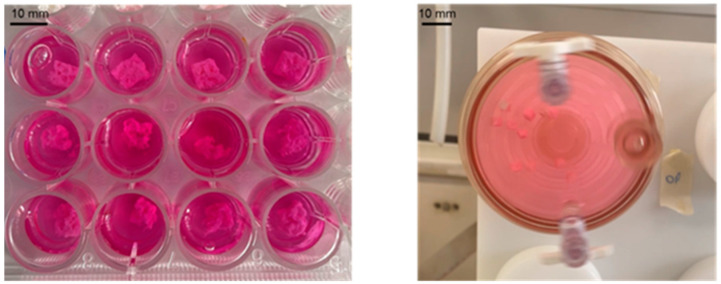
RCCS bioreactor model used in this study. Static conditions (**left** panel) and dynamic conditions (**right** panel).

Dynamic cell cultures in microgravity conditions were carried out using the Rotary Cell Culture System™ 4SCQ (Synthecon Incorporated, 8044 El Rio, Houston, TX 77054, USA). The RCCS bioreactor is shown in Figure 2; the cell culture chamber is horizontally rotated to constantly suspend the inoculated cells and any support (e.g., scaffolds) in the culture medium. Scaffolds of three different sizes (4 × 4 × 4 mm^3^, 6 × 6 × 6 mm^3^, 8 × 8 × 8 mm^3^) were tested using increasing rotational speeds (from 25 to 45 rotations per minute) to identify the optimal floating condition of the scaffolds in the culture bioreactor vessel. Proper suspension of the scaffolds is essential for establishing an effective 3D dynamic culture environment, as it allows for uniform exposure of the scaffold surfaces to nutrient flow and shear forces, which are key to promoting effective cell adhesion, proliferation, and viability. The rotational speed that ensured their optimal suspension in the medium was found to be 45 rpm, while the most suitable scaffold size for proper floating conditions was 4 × 4 × 4 mm^3^. All tests were performed using these parameters.

### 2.5. Biological Assays

For the static and dynamic cultures, SAOS-2 and U2OS were seeded on the 3D-printed scaffolds at a density of 2 × 10^4^ cell/cm^2^ and cultured in the same humidified incubator at 37 °C and 5% CO_2_, with the CO_2_ being passively maintained within the bioreactor by means of an ad hoc filter that allows for gas exchange. The scaffolds were specifically sized (4 × 4 × 4 mm^3^) to be cultured in a suspension mode into the RCCS bioreactor to balance the force acting on the scaffolds themselves (i.e., gravity, buoyancy, and centrifugal and drag forces). For the SAOS-2 and U2OS cultures in simulated microgravity, P and O scaffolds were inserted into the RCCS bioreactor chambers and the cells were directly seeded into the chamber filled with culture medium at a density of 2.0 × 10^4^ cell/cm^2^. Bubbles were removed using a syringe. On day 4, scaffolds were removed for the characterization.

#### 2.5.1. Cell Proliferation and Metabolic Activity Analysis

Cell proliferation was assessed using the Bromodeoxyuridine (BrdU) incorporation assay (Cell Proliferation Kit, Roche Diagnostics), and metabolic activity was evaluated with a colorimetric assay based on the oxidation of water-soluble tetrazolium salts (WST-1 reagent, Roche Diagnostics).

On day 4, scaffolds were removed by the RCCS bioreactor and located in a multiwall dish for assay analysis. This step ensured that the assays (BrdU, WST-1, and ELISA) specifically measured the cellular activity of scaffold-adherent cells, minimizing interference from any free-floating cells.

For the metabolic activity assay, WST-1 reagent (diluted 1:10) was added directly to the culture medium, and cells were incubated for 2 h under standard humidified conditions. Following incubation, 100 µL of the resulting supernatant was transferred to a 96-well plate, and the formation of the formazan product was quantified spectrophotometrically. For the BrdU incorporation assay, cells were exposed to 10 mM BrdU for 18 h. After incubation, the cells were fixed and treated with an anti-BrdU antibody (dilution 1:100) for 30 min at 37 °C. Subsequently, the chromogenic substrate 2,2′-azino-bis(3-ethylbenzothiazoline-6-sulfonic acid) was added and incubated for an additional 30 min. Absorbance was then measured at 450 nm using a VICTOR3 multilabel plate reader (PerkinElmer, Waltham, MA, USA).

#### 2.5.2. Quantification of TNF-α

Quantification of tumor necrosis factor-α (TNF-α) released by the cells was obtained using an ELISA development kit (PeproTech^®^ EC Ltd., London, UK) at 405 nm. Supernatants from SAOS-2 and U2OS cells grown on the scaffolds were collected at 96 h of culture, centrifuged at 1200 rpm for 5 min, and stored at −80 °C until use. The release of TNF-α was quantified in the culture medium using a human ELISA development kit (PeproTech^®^ EC Ltd., London, UK), following the manufacturer’s protocol. Cell culture supernatants and recombinant human TNF-α standards were serially diluted in PBS containing 0.05% Tween-20 and 0.1% BSA (Sigma-Aldrich^®^, Dorset, UK). Cytokine detection was carried out using a biotin–avidin system, followed by incubation with the chromogenic substrate 2,2′-azino-bis(3-ethylbenzothiazoline-6-sulfonic acid) (ABTS; Sigma-Aldrich^®^, Dorset, UK).

#### 2.5.3. Scanning Electron Microscopy (SEM) Analysis

Scanning electron microscopy (SEM) analysis was carried out to confirm the results obtained by cell growth assays. Cells cultured on the scaffolds were fixed with 4% paraformaldehyde for 10 min. After dehydration, the samples were coated with a 10 nm gold layer using an evaporation technique. SEM imaging was performed using a ZEISS SIGMA 300 field emission microscope (ZEISS, Oberkochen, Germany). Morphological evaluation was conducted at an accelerating voltage of 5 kV with secondary electron detection to visualize the cell–scaffold interactions in detail.

### 2.6. Statistical Analysis

Data are expressed as mean ± standard deviation, and MedCalc version 23.0.9 software was used for statistical analysis. Each experiment was performed in triplicate, data were analyzed with Student’s T-tests, and the significance level adopted for all analyses was *p* < 0.05.

## 3. Results

### 3.1. Scaffold Design Assessment

The nominal porosity of the scaffold models was measured by means of the “analysis” tool of the 3D modeling software Meshmixer 3.5. The P scaffold porosity was 53.77%, while the O model was characterized by a porosity level of 82.96%. The bone porosity may change depending on the skeletal site and can increase up to 90% in osteoporotic conditions [14]; in this study, the design of the O scaffolds allowed us to reach the highest porosity with thin trabeculae and an interconnected bone-like structure in the required osteoporotic range, as previously reported [15,16,17].

### 3.2. Three-Dimensional-Printed Scaffolds and Morphometric Analysis

**Figure 3:**
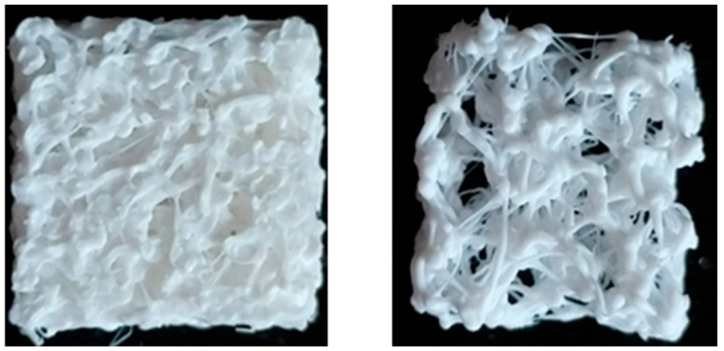
Three-dimensional-printed PLA scaffolds: physiological P model (**left** panel) and osteoporotic O model (**right** panel).

The 3D-printed PLA scaffolds are shown in Figure 3, where it is possible to observe the different microarchitectures of both the physiological and osteoporotic-like constructs.

Each scaffold was analyzed using μ-CT to study the internal microstructure and quantify the compliance of the design criteria to the printed cases. A quantitative analysis was performed analyzing µ-CT data to extract the main bone histomorphometry parameters relevant to this study (Table 2 and Table 3), which were then compared with corresponding values reported in the literature.

**Figure 4:**
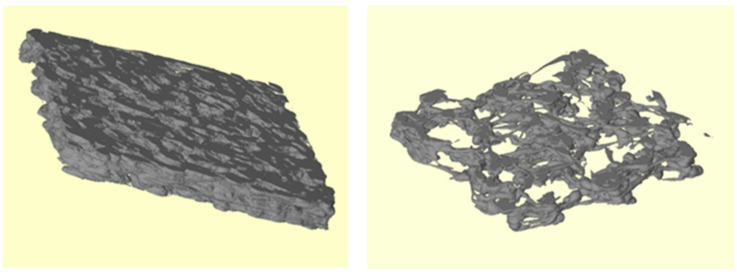
Three-dimensional images of the scaffolds obtained by μ-CT analysis: physiological P model (**left** panel) and osteoporotic O model (**right** panel).

The data obtained from the μ-CT analysis confirmed a good agreement with the literature data, especially for the osteoporotic case. This was not strictly verified for the physiological model, which can be related to the resolution limits of the 3D printer as well as to the dynamics of the molten polymer cooling during the 3D printing process, which has already been observed [22,23]. Three-dimensional images obtained through the μ-CT analysis are shown in Figure 4.

### 3.3. Cell Growth and Metabolic Activity Study in RCCS Conditions

**Figure 5:**
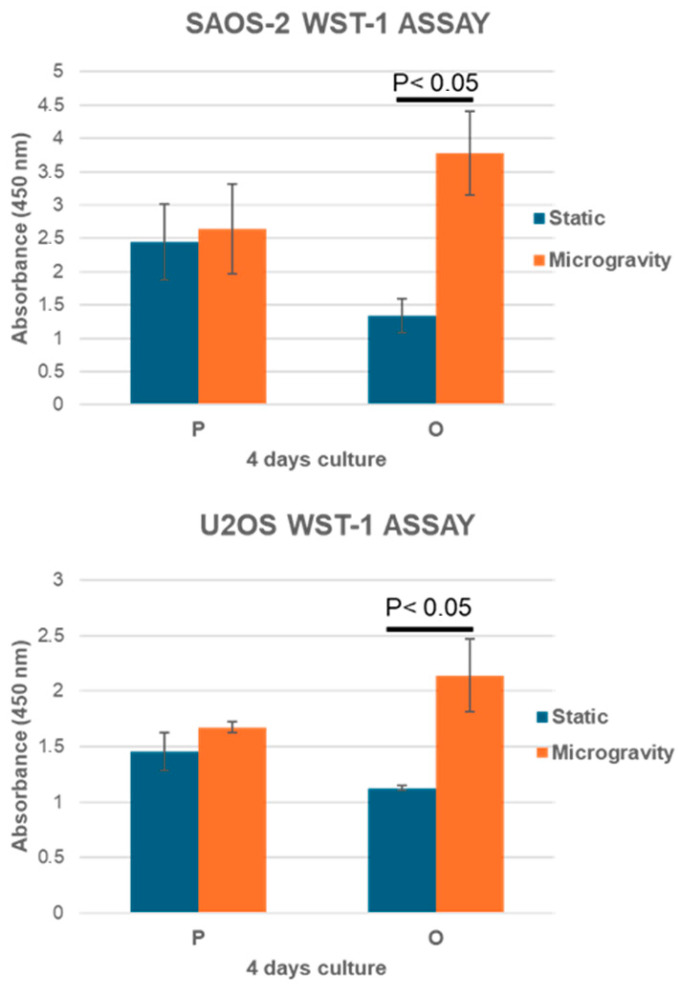
Analysis of cell metabolic activity on P and O scaffold models measured with the WST-1 assay at day 4 for both cell lines. *n* = 3, mean ± SD.

**Figure 6:**
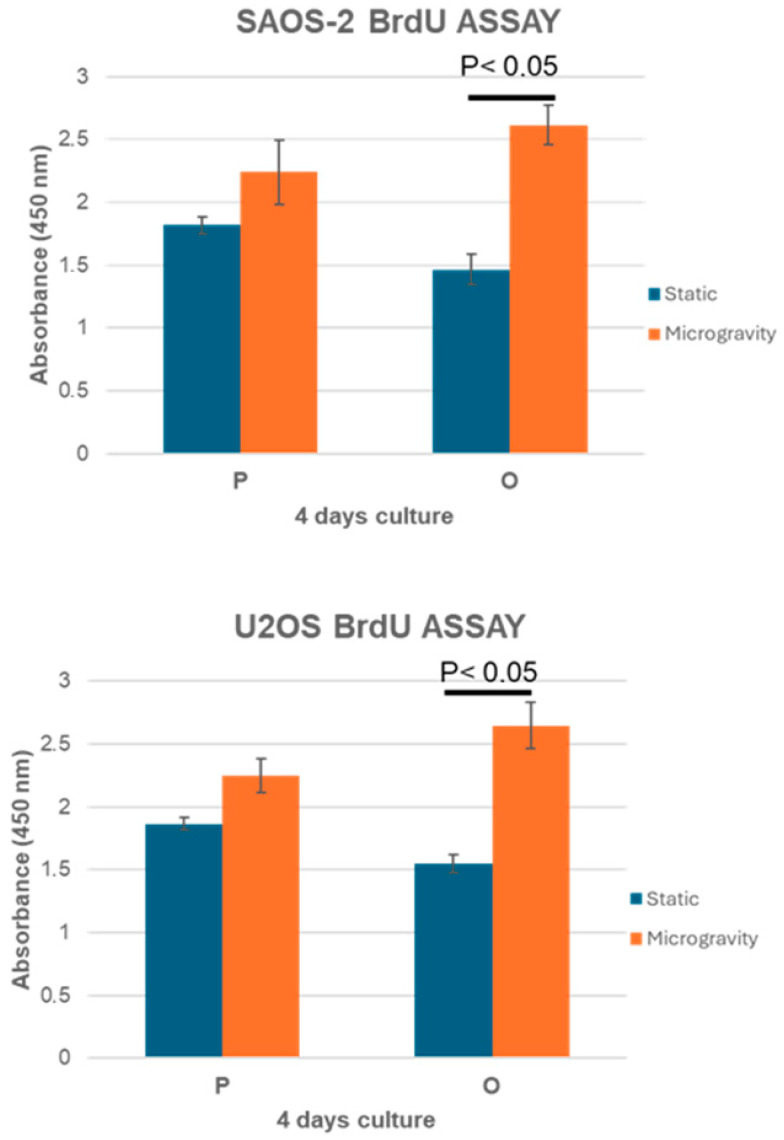
Analysis of cell growth on P and O scaffold models measured with the BrdU assay at day 4 for both cell lines. *n* = 3, mean ± SD.

Simulated microgravity was investigated to evaluate the interaction of osteoblast-like cells with the physiological and osteoporotic-like PLA scaffolds (P and O models). Two osteoblast-like cell lines, SAOS-2 and U2OS, were cultured in dynamic conditions with both PLA scaffolds using the RCCS bioreactor to investigate their capability of colonizing and growing within the constructs by means of BrdU and WST-1 colorimetric assays, in comparison to those grown in 2D static conditions. These cell lines, derived from human osteosarcomas with different differentiation levels, have been used in several studies for bone tissue engineering. SAOS-2 cells have an osteoblast phenotype with characteristics similar to human primary osteoblasts and present a high level of ALP activity and mineralization ability [24,25]. U2OS cells instead exhibit properties of mesenchymal cells [26] and have a very low level of ALP and mineralization capacity [27]. The SAOS-2 and U2OS cells cultured for 4 days in dynamic conditions showed a positive interaction with polymer substrates, which were able to support both cell growth and metabolic activity processes (Figure 5 and Figure 6).

The P scaffolds were characterized by a slight increase in cell colonization and metabolic activity in RCCS conditions, compared to the static one, while a higher growth and metabolic response were observed for the O model, in which this difference between static and dynamic cultures, obtained by BrdU and WST-1 tests, were statistically significant (*p* < 0.05). The BrdU and WST-1 assay results in a static culture revealed a decreasing trend from the less porous model to the more porous model. This trend could be due to the scaffold morphology: the P model, with an effective porosity of about 44.7%, prevents cells from settling to the bottom, allowing cells to better adhere to the scaffold surface and within the internal trabecular network, resulting in a higher cell growth after 4 days, consistent with a report by Zhou et al. [22]. Instead, the O model, due to its osteoporotic-like structure (about 80.53% of porosity), induced a lower cell interaction with the printed microarchitecture, settling at the bottom through the large pore network with a lower cell adhesion, as proven by the BrdU and WST-1 assays. In microgravity culture conditions, scaffolds and cells continuously interact, and the high porosity of the O scaffolds, by increasing the available surface area, improves the cells’ attachment. For this reason, the osteoblast-like cells (SAOS-2 and U2OS) seeded on high-porosity scaffolds (O) and cultured in dynamic conditions showed a higher cell colonization compared not only to static conditions but also to low porosity substrates (P).

### 3.4. Cell Adhesion Study in RCCS Conditions

**Figure 7:**
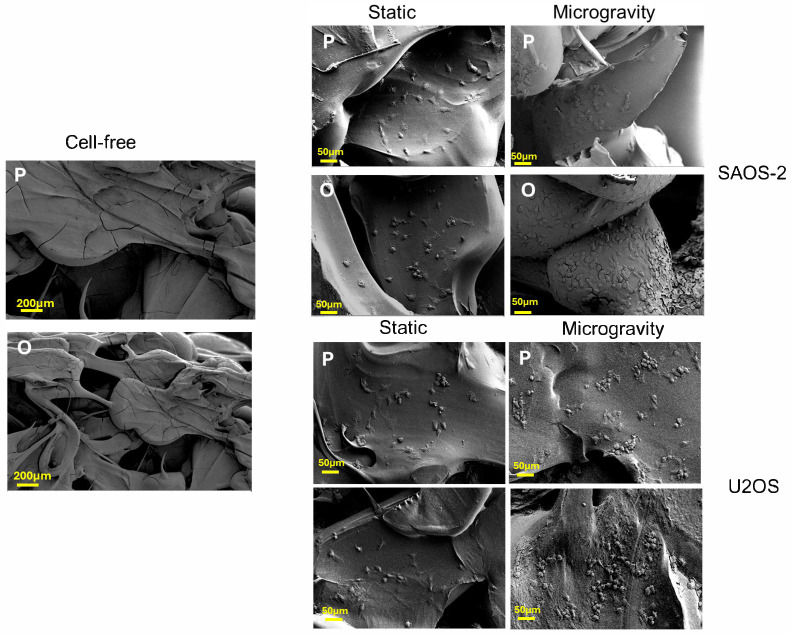
SEM analysis of cell-free scaffold and SAOS-2 and U2OS cells grown on P and O scaffold models for 4 days.

The adhesion and migration ability of cells into the scaffolds after culture in the RCCS bioreactor were studied using scanning electron microscopy (SEM) both for SAOS-2 cells, which are routinely used for investigations of osteoblast–biomaterial interactions, and for U2OS. SEM analysis allows for a detailed visualization of the scaffold’s structure and the spatial distribution of cells within it. The images reported in Figure 7 confirmed a random porous microarchitecture. SAOS-2 and U2OS cells typically have polygonal, spindle-shaped, and fibroblast-like morphology (Figure S1) and are strongly adherent to the scaffold’s surfaces, demonstrating their ability to colonize and penetrate the scaffold. This behavior was more pronounced in samples cultured under dynamic (microgravity) conditions compared to static ones. In particular, for both cells, SEM images revealed a noticeably higher cell density and more extensive surface coverage under dynamic conditions, suggesting improved adhesion and migration into the scaffold structure. In contrast, under static conditions, the cells appeared less uniformly distributed and in a lower density, indicating that the dynamic culture environment provided by the RCCS bioreactor enhances cell–scaffold interactions. These comparative observations, consistently confirmed for both SAOS-2 and U2OS cells, highlight the positive effect of a dynamic culture in the RCCS bioreactor, which creates a more favorable 3D environment for cellular interaction with the scaffold, supporting improved colonization.

### 3.5. TNF-α Secretion Analysis

**Figure 8:**
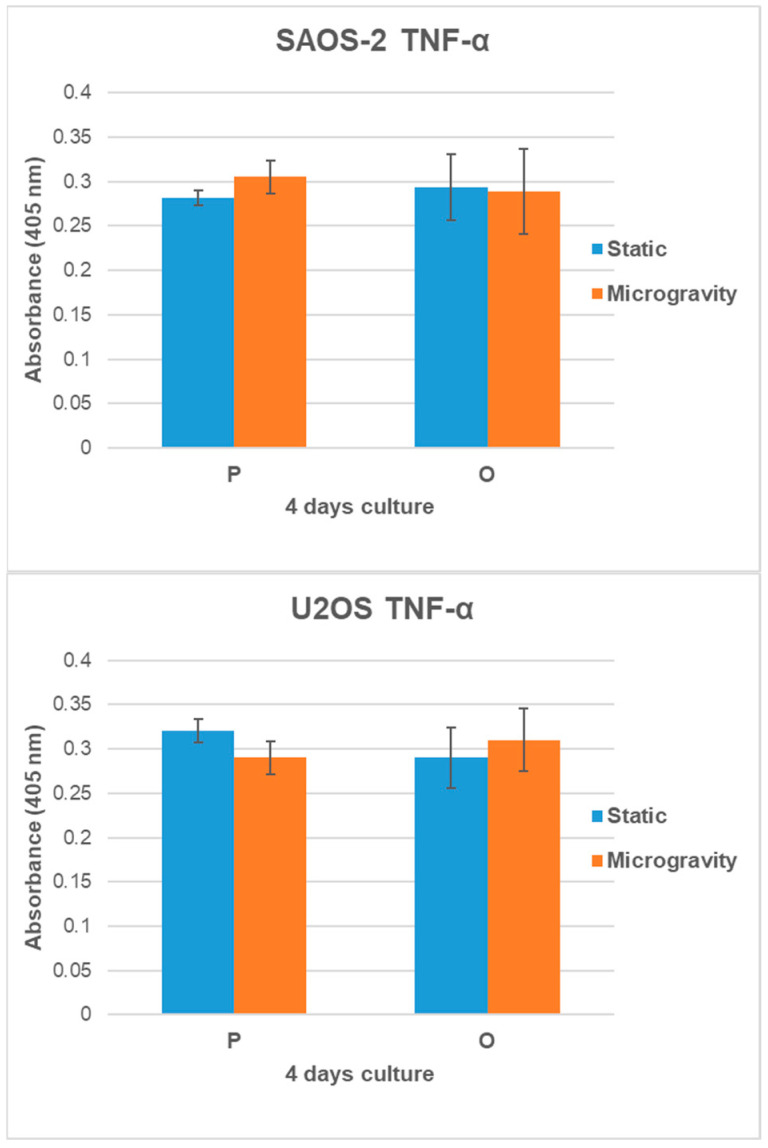
SAOS-2 and U2OS TNF-α concentration analysis grown on P and O scaffold models. *n* = 3, mean ± SD.

The results of cell growth, metabolic activity, and cell adhesion confirmed the biocompatibility of the analyzed scaffolds and the dynamic bioreactor. To further support these findings, and as a preliminary step to evaluate the potential effects of the combination of scaffolds and RCCS culture conditions, the pro-inflammatory TNF-α levels secreted by SAOS-2 and U2OS cells, grown on P and O scaffolds, were evaluated using an ELISA assay. TNF-α was selected as an initial inflammatory marker due to its well-established role in osteoimmune signaling. A substantial body of evidence identifies TNF-α as a key pro-inflammatory cytokine in osteoimmune interactions, promoting osteoclastogenesis, suppressing osteoblast activity, and facilitating bone resorption under inflammatory conditions [28,29]. Figure 8 shows the TNF-α concentration, measured as the absorbance level at 405 nm for all conditions studied at 4 days of culture.

Evaluating the secretion levels of TNF-α, no experimental group produced detectable amounts of this proinflammatory factor: the concentration in the samples was undetectable compared to the measured absorbance and the standard curve of TNF-α concentration. This outcome states that the scaffolds do not induce an inflammatory response after 96 h of culture, further confirming the biocompatibility of the substrates and the dynamic platform.

## 4. Discussion

### 4.1. Bioreactor System and Scaffold Design

Three-dimensional cell culture systems represent a major advancement in biomedical research, providing an improved in vitro model to study cellular behavior in a more physiologically relevant microenvironment. In addition, their integration with dynamic bioreactors has opened new opportunities in tissue engineering and regenerative medicine [30]. In this study, we investigated the interaction between osteoblast-like cells and 3D-printed scaffolds that mimic physiological and osteoporotic bone structures under simulated microgravity conditions. Bone remodeling is significantly affected by microgravity, inducing a faster resorption process than ossification [31]. Gravity and mechanical stimuli, e.g., tensile and compressive stresses, fluid-exerted shear stress, and hydrostatic pressure, concur to sustain the maintenance of healthy tissues and cells [32]; these input conditions need to be investigated in detail to develop specific countermeasures. In this regard, additive manufacturing may be a suitable technology to prepare tissue replicas to foster bone research in 3D conditions. The possibility to modulate the architecture of the bone microenvironment, in terms of pore size, porosity, and trabecular arrangement, can be promptly realized to test the biological output of different bone-related cells [33]. To promote an advancement in the field and perform in vitro analyses resembling bone physiopathology, PLA bone scaffolds with increasing porosity from a low physiological value to an osteoporotic range were designed and 3D printed using fused deposition modeling. The morphometric characteristics of the P model, measured using μ-CT, were slightly different from the literature data with respect, in this case, to the physiological range of the human proximal ulna [18]; these differences can be ascribed to the resolution limit of the 3D printer. Regarding the design values selected for the O model, these were set to create a biomimetic trabecular architecture whose structural parameters fall within the osteoporotic range, referred to as the femoral head [14,17,19,20,21], which is a skeletal site dramatically prone to osteoporosis both under normal Earth gravity conditions and in weightlessness.

### 4.2. Cellular Responses and Biocompatibility

Regarding biological assessment, the results showed that both the osteoblast-like cell lines used positively interacted with all PLA substrates [34], demonstrating that the fabricated scaffolds and the dynamic cell culture system studied were biocompatible and able to support cell growth, adhesion, and metabolic activity without an inflammatory response, as proven by BrdU, WST-1, and ELISA assays.

SAOS-2 and U2OS cells, derived from human osteosarcomas with different levels of differentiation, represent well-established models for bone tissue engineering investigations. They can be used for studying the osteogenesis process and how it could be affected by different treatments. SAOS-2 cells have the capability to produce a mineralized matrix and express osteocyte marker genes, as well as the potential to mimic the interaction between primary human osteoblasts and biomaterials [13]. U2OS has a lower osteoblastic differentiation level and exhibits mesenchymal cell properties [26]. For this reason, both cell lines were chosen in this work as we aimed to confirm our results using two distinct cell lines with different differentiation levels, ensuring the robustness and reproducibility of the findings across different cellular contexts. We aimed to confirm our results using two distinct cell lines with different differentiation levels, ensuring the robustness and reproducibility of the findings across different cellular contexts. This comparative approach provided deeper insights into how scaffold properties and bioreactor conditions influence cellular behavior.

The cells were cultured for a short period of four days across the different experimental conditions, as the primary goal was to examine the early phase of cell–scaffold interactions. Specifically, this study aimed to assess how scaffold microarchitecture and dynamic fluid conditions influence initial cellular responses such as adhesion, viability, and metabolic activity. These preliminary findings were instrumental in validating the proposed in vitro platform, which combines 3D-printed scaffolds with a rotary dynamic culture system. Establishing this integrated setup represents a foundational step for future long-term studies focused on osteogenic differentiation and bone tissue remodeling in dynamic environments that more closely replicate physiological conditions.

The results obtained using BrdU and WST-1 assays highlighted that scaffold morphology, mainly porosity, affects cell adhesion and growth when comparing static and dynamic cell culture conditions. This effect is greater in dynamic conditions because the 3D culture environment and the fluid motion support cells to interact with a larger surface area of the substrate, thus amplifying the effect of the different scaffold porosity on cell adhesion and viability [35,36]. Particularly, the best cell colonization and metabolic response occurs for cells cultured within the O model in the RCCS bioreactor, suggesting that the combination of this culture condition and the most porous scaffold (O model) provides the best cell–scaffold interaction, probably due to a suitable fluid flow condition established within the substrate framework. This finding underscores the importance of dynamic culture conditions, which allow cells to interact with a larger surface area, improving attachment and viability.

The absence of detectable TNF-α secretion by cells cultured under both static and dynamic conditions on the scaffolds confirms the system’s biocompatibility, as already demonstrated by the results on cell growth, adhesion, and metabolic activity, and further suggests a lack of inflammatory response [33,36]. TNF-α is a key mediator of early osteoimmune activation; however, it represents only one element of the broader inflammatory cascade. While the current findings provide encouraging evidence of scaffold and bioreactor biocompatibility, the absence of TNF-α alone offers only a preliminary indication of the system’s inflammatory behavior and should be interpreted within this limited context. For a more comprehensive assessment of immunocompatibility in dynamic 3D culture systems, future studies should include additional markers such as IL-6 and IL-1β, and possibly co-culture models.

### 4.3. Implications for Bone Tissue Engineering Research

The combination of additive manufacturing and rotary cell culture systems enabled the creation of biomimetic platforms with precise control over scaffold microarchitecture, offering an innovative system to study bone remodeling and for tissue engineering applications. The advantages of 3D cell culture models are particularly evident when studying bone tissue engineering strategies. Traditional 2D cell cultures fail to replicate the complex structural and mechanical properties of bone, limiting their relevance in preclinical research. The RCCS bioreactor system, by mimicking a low-shear microgravity environment, provides a more biomimetic approach for investigating bone cell behavior, particularly in the context of osteoporosis and microgravity-induced bone loss. The enhanced nutrient diffusion and dynamic fluid conditions within the RCCS further contribute to a more physiologically relevant culture environment. At the same time, it should also be underlined that the dynamic conditions established within a rotating bioreactor lead to a non-uniform flow regimen, which might affect cell response in terms of shear stress and nutrition/waste transfer that is locally experienced (e.g., center vs. periphery of the vessel). However, the collected results, supported by additional studies, can have important implications for both space research and terrestrial biomedical applications. The ability to simulate bone loss conditions in microgravity offers a valuable tool for studying the mechanisms of osteoporosis and developing countermeasures. Additionally, the use of high-porosity scaffolds in a dynamic 3D culture system may provide novel insights into optimizing scaffold design for bone tissue engineering. Future research should focus on refining scaffold materials and architecture, as well as investigating the role of interstitial fluid dynamics in regulating osteogenic activity. Clearly, a refinement of the biological protocol is also necessary to evaluate the cell response to longer conditioning periods in the RCCS and to specifically address microgravity-induced alterations in bone remodeling by means of specific assays, including osteogenic marker expression analysis. By leveraging advanced 3D cell culture systems and dynamic bioreactors, this research may pave the way to bridge the gap between in vitro models and in vivo applications. The integration of biomimetic scaffolds with simulated microgravity conditions presents a promising strategy for improving bone tissue engineering approaches, ultimately contributing to the development of more effective therapies for osteoporosis and other degenerative bone disorders.

## 5. Conclusions

Data from the biological assays of this work show that 3D-printed PLA scaffolds with an osteoporotic bone-like structure, rather than worsening cell growth, improve cell response when cultured in a RCCS bioreactor to simulate weightlessness. This outcome is probably related to the fluid dynamics established within the scaffold, which contributes to enhancing the cell response. A 3D-printed osteoporotic-like scaffold may be regarded as a potential model to assess possible countermeasures to stimulate and improve cell activity, simulating bone disorders when exposed to dynamic culture conditions. A RCCS bioreactor can represent a suitable in vitro set-up for this aim thanks to the 3D culture capability, fluid dynamics, nutrient transfer, and gas exchange that allow us to perform more effective and specific studies with respect to the conventional static approach. These results need to be further refined, as microgravity-simulating bioreactors and osteoporotic bone models could be used to better analyze the effects of fluid flow in the osteo-activity regulation as a means to design a potential protocol to treat microgravity and/or age-related osteoporosis. Biological data also suggest that it is probably feasible to achieve a fluid condition suitable for osteogenic activities even in bone tissues with higher porosity, supporting future research projects that would be focused on the role of interstitial fluid flow in osteoporotic bone.

## Figures and Tables

**Table 1:** Parameters used for CAD modeling: pore dimension (PD), pore spacing (PS), trabecular thickness (TT), and trabecular spacing (TS).

Sample	PD (µm)	PS (µm)	TT (µm)	TS (µm)
P	700	700	200	600
O	800	500	150	800

**Table 2:** Morphometric parameters for the physiological P scaffold.

Parameters	μ-CT Data	Literature Range	Reference
Trabecular thickness [µm]	258	310–490	[18]
Trabecular spacing [µm]	284	410–850	[18]
Porosity (%)	44.7	34–78	[18]

**Table 3:** Morphometric parameters for the pathological O scaffold.

Parameters	μ-CT Data	Literature Range	Reference
Trabecular thickness [µm]	215	90–230	[19]
Trabecular spacing [µm]	691	680–750	[14,19,20,21]
Porosity (%)	80.5	78–88	[17,19,20,21]

## Data Availability

The original contributions presented in this study are included in the article; further inquiries can be directed to the corresponding authors.

## Supplementary Materials

The following supporting information can be downloaded at: https://www.mdpi.com/article/10.3390/jfb16080271/s1, Figure S1: Higher-magnification SEM images of SAOS-2 and U2OS cells grown on P and O scaffold models for 4 days. The cells have the typical polygonal, spindle-shaped, and fibroblast-like morphology and are strongly adherent to the scaffolds.
