# Supplementary material for: Three-Dimensional-Printed Biomimetic Scaffolds for Investigating Osteoblast-Like Cell Interactions in Simulated Microgravity: An In Vitro Platform for Bone Tissue Engineering Research
Source: J Funct Biomater. 2025 Jul 24;16(8):271. doi: 10.3390/jfb16080271 (PMC12387348; doi:10.3390/jfb16080271)
Supplement: Supplementary file 1 [file jfb-16-00271-s001.zip › jfb-3703348-supplementary.pdf]

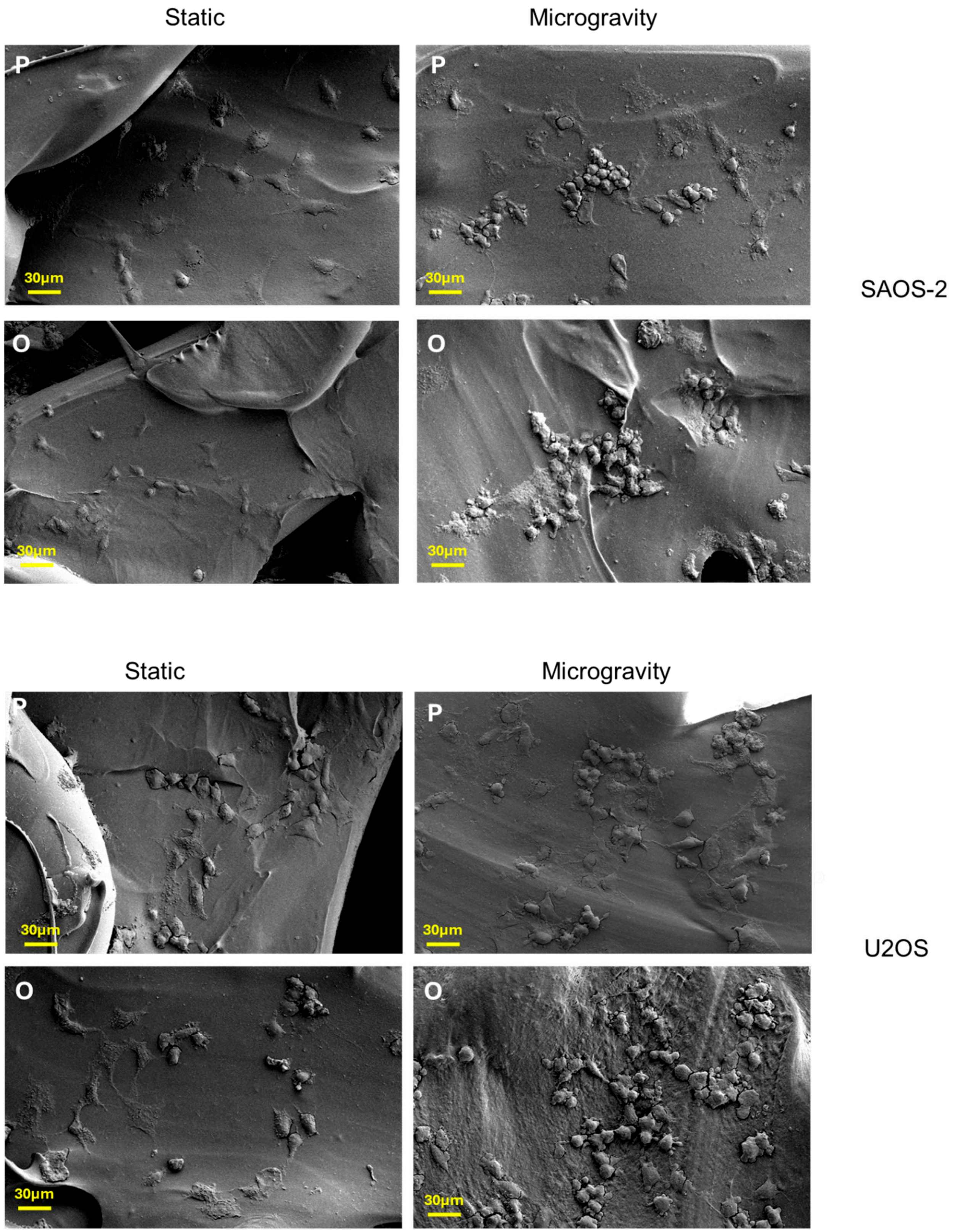

Figure S1. Higher-magnification SEM images of SAOS-2 and U2OS cells grown on P and O scaffold models for 4 days. The cells have the typical polygonal, spindle-shaped, and fibroblast-like morphology and are strongly adherent to the scaffolds.
